# A Single Amino Acid Dictates Protein Kinase R Susceptibility to Unrelated Viral Antagonists

**DOI:** 10.1371/journal.ppat.1005966

**Published:** 2016-10-25

**Authors:** Kathryn S. Carpentier, Nicolle M. Esparo, Stephanie J. Child, Adam P. Geballe

**Affiliations:** Departments of Microbiology and Medicine, University of Washington, Seattle Washington, and Divisions of Human Biology and Clinical Research, Fred Hutchinson Cancer Research Center, Seattle, Washington; Blumburg Institute, UNITED STATES

## Abstract

During millions of years of coevolution with their hosts, cytomegaloviruses (CMVs) have succeeded in adapting to overcome host-specific immune defenses, including the protein kinase R (PKR) pathway. Consequently, these adaptations may also contribute to the inability of CMVs to cross species barriers. Here, we provide evidence that the evolutionary arms race between the antiviral factor PKR and its CMV antagonist TRS1 has led to extensive differences in the species-specificity of primate CMV TRS1 proteins. Moreover, we identify a single residue in human PKR that when mutated to the amino acid present in African green monkey (Agm) PKR (F489S) is sufficient to confer resistance to HCMV_TRS1_. Notably, this precise molecular determinant of PKR resistance has evolved under strong positive selection among primate PKR alleles and is positioned within the αG helix, which mediates the direct interaction of PKR with its substrate eIF2α. Remarkably, this same residue also impacts sensitivity to K3L, a poxvirus-encoded pseudosubstrate that structurally mimics eIF2α. Unlike K3L, TRS1 has no homology to eIF2α, suggesting that unrelated viral genes have convergently evolved to target this critical region of PKR. Despite its functional importance, the αG helix exhibits extraordinary plasticity, enabling adaptations that allow PKR to evade diverse viral antagonists while still maintaining its critical interaction with eIF2α.

## Author Summary

Cytomegaloviruses (CMVs) are highly species-specific, but the host factors that prevent replication in heterologous species are largely unknown. Based on data indicating that the broadly-acting host antiviral factor protein kinase R (PKR) has diversified rapidly during evolution, we hypothesized that PKR may contribute to cross-species barriers to CMV replication. In support of this hypothesis, we find that primate CMVs differ in their ability to antagonize PKRs from different primates. By leveraging these differences, we identified a single amino acid at codon 489 in human PKR that dictates PKR susceptibility to the human CMV PKR antagonist, HCMV_TRS1_. This amino acid is positioned within a helix that mediates the critical interaction between PKR and its downstream substrate eIF2α. Despite this seemingly important structural role, human PKR is highly tolerant of amino acid substitutions at position 489, allowing it the flexibility to adapt in order to evade viral antagonists without disrupting its antiviral activity. Remarkably, position 489 also dictates PKR sensitivity to the entirely unrelated poxvirus-encoded PKR antagonist, K3L. Thus, mutations driven by one virus can impact the host’s sensitivity to unrelated viral antagonists, illustrating the multilateral nature of the host-viral “arms-races” between viruses and broadly acting antiviral host defenses.

## Introduction

HCMV is a ubiquitous virus that persists for the lifespan of the infected host, highlighting its ability to evade host defenses [1]. While most infections are asymptomatic, HCMV causes life-threatening diseases in immunocompromised patients and is the most frequent congenital viral infection in developed countries, leading to permanent neurological deficits in thousands of newborns each year [2]. Despite its success in spreading throughout the human population, HCMV is unable to cross species barriers. Genomic analyses have demonstrated that CMVs have been co-speciating with their hosts for ~80 million years [3,4]. Through this process, each CMV has specifically adapted to its cognate host and in doing so, diverged from closely related CMV species. Among the many factors that may contribute to cross-species barriers to infection, cell-intrinsic immune factors likely play a central role because the selective pressure imposed by viral antagonists has driven their rapid evolution. Support for this arms race paradigm comes from computational and functional studies that demonstrate ongoing reciprocal innovation by host and viral factors at host:virus interfaces [5,6]. The millions of years of shared evolutionary history between CMVs and their hosts provide an invaluable model for investigating the consequences of host-virus arms races.

Protein Kinase R (PKR) is a broadly acting restriction factor that phosphorylates the translation initiation factor eIF2α in response to cytoplasmic double-stranded RNA (dsRNA), resulting in a block to translation initiation and viral replication [7]. The importance of PKR in the host cell’s anti-viral arsenal is highlighted by the presence of PKR antagonists in many virus families [8,9]. Furthermore, deletion of PKR antagonists renders many viruses replication deficient [10–15], demonstrating that PKR poses a strong molecular barrier to viral replication. To overcome the onslaught of diverse viral antagonists, PKR has had to continually adapt while still being constrained by the need to maintain its critical interactions with dsRNA and eIF2α. Consistent with this perspective, evolutionary analyses have identified dramatic episodes of positive selection in PKR during primate evolution [16,17]. Thus, we first leveraged the long co-evolutionary history of CMVs and their hosts to investigate how the rapid evolution of PKR has impacted the evolution of the CMV-encoded PKR antagonist TRS1.

## Results

### Primate cytomegaloviruses have evolved species-specific differences in PKR antagonism

**Fig 1:**
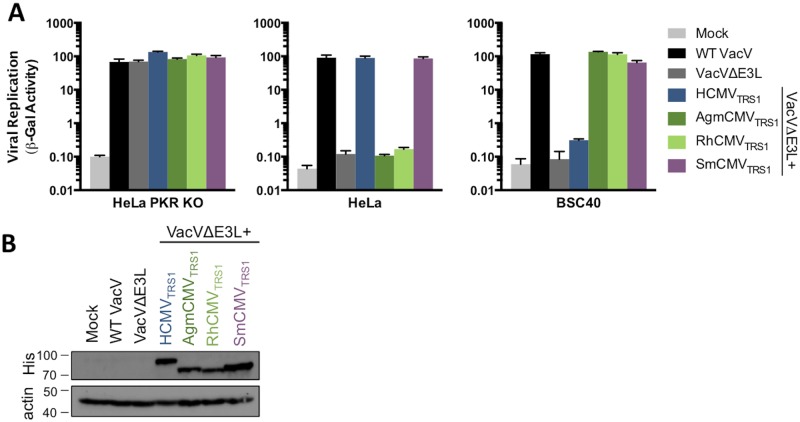
Species-specific differences in primate CMV TRS1 PKR antagonism. (A) HeLa PKR KO, HeLa (human), or BSC40 (Agm) cells were mock infected or infected (MOI 0.1) with WT VacV, VacVΔE3L, or VacVΔE3L recombinants containing HCMV_TRS1_, AgmCMV_TRS1_, RhCMV_TRS1_, or SmCMV_TRS1_. At 48 hpi, viral replication was quantified by measuring β-gal activity (mean ± s.d., n = 3). Data are representative of three independent experiments. (B) His-tagged TRS1 constructs were detected in lysates of the infected HeLa PKR KO cells from (A) by western blotting. TRS1 size variation is expected based on differences in coding length.

To determine whether the evolutionary divergence of PKR in primates has affected the ability of CMVs to antagonize PKR, we used a recombinant VacV system to readily test TRS1 alleles from several primate CMV species. The VacVs used in these studies were engineered to express *lacZ*, allowing us to measure β-gal activity as a proxy for viral replication as there is strong correlation between β-gal activity and viral titers (S1 Fig). This recombinant VacV system takes advantage of the fact that wild type VacV (WT VacV) replicates well in a broad range of primate cells, including human (HeLa) and Agm (BSC40) cells, while deletion of the PKR antagonist E3L (VacVΔE3L) markedly reduces replication [14] (Fig 1A). PKR activity is responsible for the replication blockade in HeLa cells, as knocking out PKR by CRISPR/Cas9 gene editing (HeLa PKR KO) completely rescued VacVΔE3L replication. To evaluate whether the CMV TRS1 proteins can antagonize human or Agm PKR, we recombined four CMV *TRS1* genes from species that infect hominoids, Old World monkeys, and New World monkeys (HCMV, African green monkey CMV (AgmCMV), Rhesus CMV (RhCMV)), and Squirrel monkey CMV (SmCMV)) into VacVΔE3L and evaluated their ability to rescue replication in human and Agm cells. All viruses replicated well in the HeLa-PKR KO cells (Fig 1A) and expressed comparable levels of the TRS1 proteins (although in this particular experiment SmCMV_TRS1_ expression was slightly higher) (Fig 1B). However, we observed species-specific differences in viral replication in the human and Agm cell lines. Consistent with previous findings, VacVΔE3L+HCMV_TRS1_ replicated well in human but not Agm cells [18]. Conversely, AgmCMV_TRS1_ and RhCMV_TRS1_ rescued VacVΔE3L in Agm but not human cells. Surprisingly, SmCMV_TRS1_ rescued replication in both human and Agm cells, indicating that it is more broadly acting. These results suggest that evolutionary pressures have had a substantial impact on the CMV *TRS1* genes, resulting in species-specific differences in their ability to block host defenses necessary to rescue VacVΔE3L replication.

**Fig 2:**
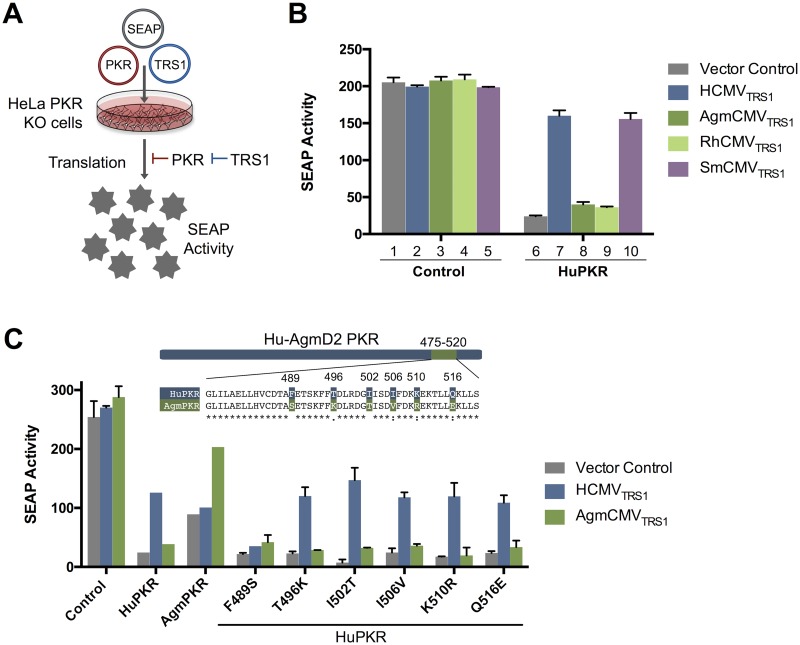
Species-specific differences in primate CMV TRS1 PKR antagonism map to a single amino acid. (A) Schematic representation of the SEAP assay. Transfection of PKR leads to decreased activity of a co-transfected reporter construct expressing SEAP. This PKR-driven repression can be counteracted by co-transfection of a functional TRS1 antagonist, resulting in a rescue of SEAP activity. (B) The SEAP assay recapitulates species-specific differences in HuPKR antagonism by TRS1 alleles. HeLa PKR KO cells were co-transfected with a SEAP reporter plasmid along with either a control vector or HuPKR and the indicated TRS1 alleles or a vector control. At 48 h post-transfection, SEAP activity in the medium was measured (mean ± s.d., n = 2). Data are representative of three independent experiments. (C) A single amino acid change, F489S, confers resistance to HCMV_TRS1_. Point mutants were generated in HuPKR to introduce the six AgmPKR-specific residues that differ between HuPKR and AgmPKR within the region spanning codons 475 to 520, shown in the alignment. The ability of the point mutants to antagonize HuPKR was evaluated as described in (B) (mean ± s.d., n = 2). Data are representative of three independent experiments.

We hypothesized that the differences in the ability of *TRS1* genes to rescue VacVΔE3L in human and Agm cells are due to differing abilities to antagonize human vs. Agm PKR. To test this, we utilized an assay in which expression of PKR inhibits translation of a co-transfected secreted embryonic alkaline phosphatase (SEAP) reporter gene [17,19] (Fig 2A). Co-transfection of a functional PKR antagonist reverses, at least in part, the inhibitory effect of PKR, leading to an increase in reporter gene expression. We co-transfected HeLa PKR KO cells with plasmids expressing SEAP along with HuPKR or with a control plasmid and a panel of TRS1 antagonists or a vector control. In the absence of any antagonist, transfection of the HuPKR expression plasmid reduced SEAP expression (Fig 2B, bars 1 vs 6). Co-transfection of HCMV_TRS1_ or SmCMV_TRS1_ each lessened the inhibitory effect of HuPKR (bars 7 and 10), while co-transfection of AgmCMV_TRS1_ or RhCMV_TRS1_ had little effect (bars 8 and 9). In contrast, in cells transfected with Agm PKR, AgmCMV_TRS1_ rescued reporter activity relative to the vector control while HCMV_TRS1_ did not (Fig 2C, S2A and S2B Fig), consistent with what was observed with VacVΔE3L rescue in Agm cells (Fig 1A). These results substantiate the co-evolutionary history of CMVs with their hosts, during which specific adaptations to their cognate PKR limited hominoid and Old World monkey CMVs ability to restrict more distant PKR alleles.

### A single amino acid dictates human PKR susceptibility to HCMV_TRS1_ activity

These functional differences are likely explained by species-specific changes in the binding interface of PKR and the CMV TRS1 proteins. However, the location of this interface on PKR is unknown. As an alternative to blind approaches like alanine scanning and random mutagenesis, we leveraged the power of the species-specific differences in TRS1 activity to precisely map the PKR:TRS1 interface. We generated chimeras between the susceptible HuPKR allele and the resistant AgmPKR allele. The 98 amino acids that differ between HuPKR and AgmPKR are scattered throughout the protein, as are the sites that were previously found to be evolving under positive selection in primates [16], making it difficult to predict which region(s) is likely to be responsible for their differential sensitivity to HCMV_TRS1_. Therefore, we first made chimeras with a break point within the linker region near the middle of PKR and found that the species-specificity of HCMV_TRS1_ and AgmCMV_TRS1_ mapped to the C-terminal half of PKR (S2A Fig). Further subdivision identified a small region (codons 475–520, designated region D2) within Agm PKR which when introduced into HuPKR was sufficient to confer resistance to HCMV_TRS1_ (S2B Fig). Six amino acids within this region differ between HuPKR and AgmPKR. To determine whether any of these differences alone is necessary for sensitivity to HCMV_TRS1_, we generated point mutants at each of these sites by introducing the amino acid present in AgmPKR into HuPKR. Strikingly, mutating position 489 of HuPKR from phenylalanine to serine (F489S) was sufficient to confer resistance to HCMV_TRS1_, while the other five point mutants had no effect (Fig 2C). Thus, this system allowed us to rapidly identify position 489 as a critical species-specific determinant of sensitivity to HCMV_TRS1_.

### HuPKR F489S confers resistance to HCMV_TRS1_ in the context of viral replication

**Fig 3:**
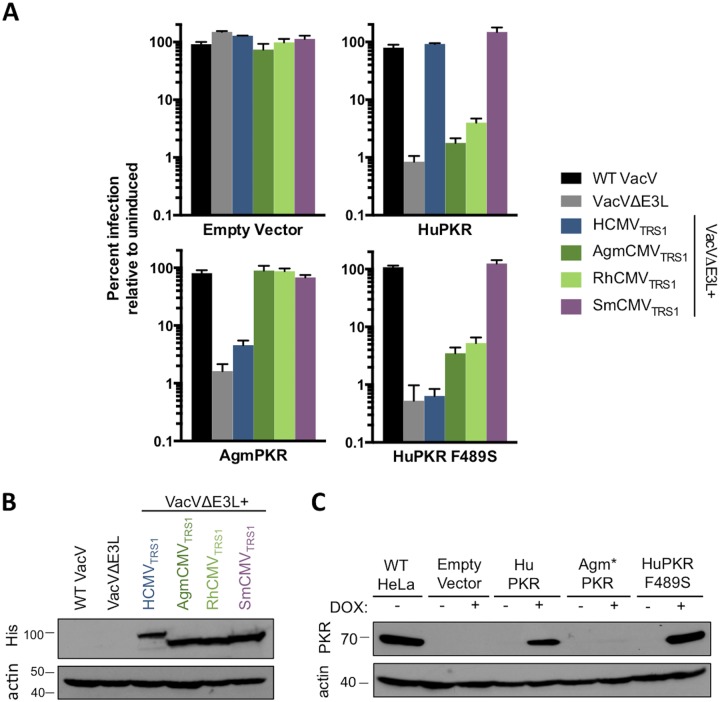
HuPKR F489S confers resistance in the context of viral replication. (A) Triplicate wells of HeLa PKR KO cells inducibly expressing the indicated PKR variants were treated +/- doxycycline and infected (MOI 0.1) with a panel of VacVs. At 48 hpi, viral replication was quantified by measuring β-gal activity and is reported as percent replication in doxycycline treated cells relative to replication in the same cells without induction of PKR expression (mean ± s.d., n = 3). Data are representative of three independent experiments. (B) His-tagged TRS1 constructs were detected in lysates of the infected empty vector cells from (A) by western blotting. TRS1 size variation is expected based on differences in coding length. (C) PKR expression in lysates of mock-infected cells from (A) was evaluated by western blotting. *AgmPKR does not cross react with the antibody used.

We next wished to test the impact of this mutation in the context of a complete viral infection by challenging viruses with heterologous PKRs. Unfortunately, this approach has been difficult to establish because PKR overexpression inhibits cell growth. To circumvent this problem, we stably transduced PKR knockout cells with heterologous PKR genes (HuPKR, AgmPKR, HuPKR F489S, or the empty vector) under the control of a doxycycline-inducible promoter. These cells were then used to assess the replication profiles of our recombinant VacVs. All of the viruses replicated well in the control empty vector cell line regardless of doxycycline induction (Fig 3A), and TRS1 proteins were expressed to similar levels in these cells (Fig 3B). Upon induction, the HuPKR and HuPKR F489S cell lines expressed PKR to levels comparable to that observed in wild-type HeLa cells (Fig 3C). Although we could not assess expression of AgmPKR as it does not cross-react with the PKR antibody, the fact that the AgmPKR cell line restricted VacVΔE3L replication after induction of PKR by addition of doxycycline strongly suggests that AgmPKR was expressed in these cells. As expected, WT VacV was able to overcome PKR antiviral activity in each of these cell lines, while VacVΔE3L did not replicate well in any (Fig 3A). In the HuPKR- and AgmPKR-expressing cell lines, the replication profiles of the panel of TRS1-expressing viruses mirrored those observed in human (HeLa) and Agm (BSC40) cell lines (Figs 1A and 3A). Importantly, the cell line expressing HuPKR F489S restricted VacVΔE3L+HCMV_TRS1_, demonstrating that this mutation renders HuPKR resistant to HCMV_TRS1_ activity in the context of viral infection. RhCMV_TRS1_ and AgmCMV_TRS1_ were also unable to antagonize HuPKR F489S efficiently and rescue replication, suggesting that mutating position 489 of HuPKR to the AgmPKR variant is not sufficient for conferring sensitivity to these Old World monkey CMV TRS1 proteins (Fig 3A). Interestingly, SmCMV_TRS1_ rescued VacVΔE3L replication even in cells expressing HuPKR F489S, consistent with its broad PKR inhibitory activity (Fig 3A).

**Fig 4:**
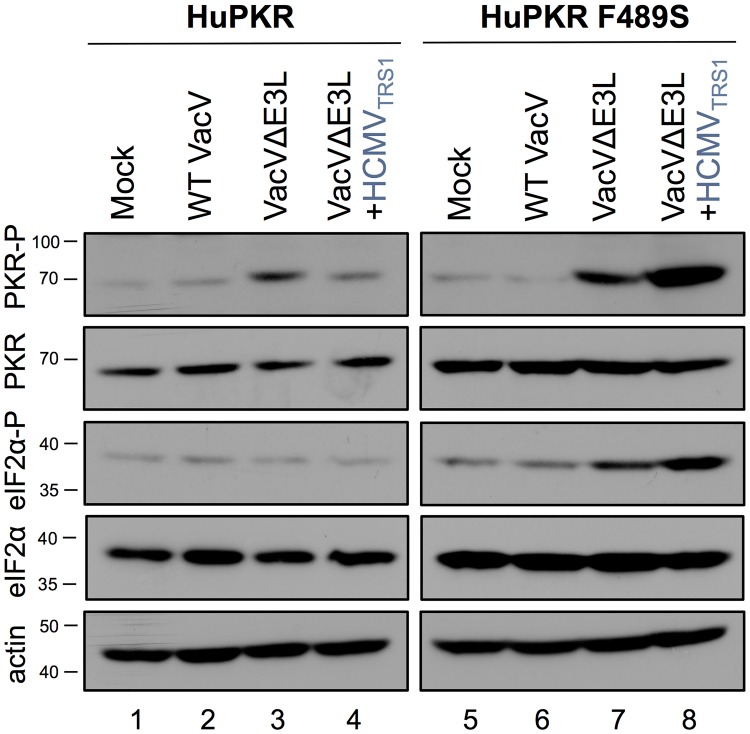
The PKR pathway is activated in HuPKR F489S cells after infection with VacVΔE3L+HCMV_TRS1_. HeLa PKR KO cells with stably integrated HuPKR or HuPKR F489S were induced with doxycycline to express PKR and 24 hours later mock-infected or infected at an MOI of 3 with the indicated viruses. At 6 hpi, cells were lysed and levels of PKR-P, total PKR, eIF2α-P, total eIF2α and actin were evaluated by western blotting.

The inability of VacVΔE3L+HCMV_TRS1_ to replicate in HuPKR F489S cells strongly suggests that HCMV_TRS1_ is unable to prevent activation of HuPKR F489S. To test this, we evaluated the levels of phosphorylated PKR and eIF2α following infection. As expected, PKR phosphorylation was observed in response to VVΔE3L infection in both HuPKR and HuPKR F489S cells, demonstrating that activation of the PKR pathway had been initiated (Fig 4). While we did not observe increased eIF2α-P levels in response to VVΔE3L infection in the HuPKR cell line in this experiment, we did observe increased eIF2α-P in the HuPKR F489S cells. Consistent with previous findings, VacVΔE3L+HCMV_TRS1_ was able to block phosphorylation of PKR and eIF2α in HuPKR cells [18]. However, robust phosphorylation of both PKR and eIF2α was observed in HuPKR F489S cells in response to VacVΔE3L+HCMV_TRS1_ infection (Fig 4, lanes 4 vs 8). Thus, consistent with the replication data (Fig 3A), HCMV_TRS1_ prevents activation of the PKR pathway in HuPKR cells but not in HuPKR F489S cells.

### Mutating position 489 disrupts HCMV_TRS1_ binding to HuPKR

**Fig 5:**
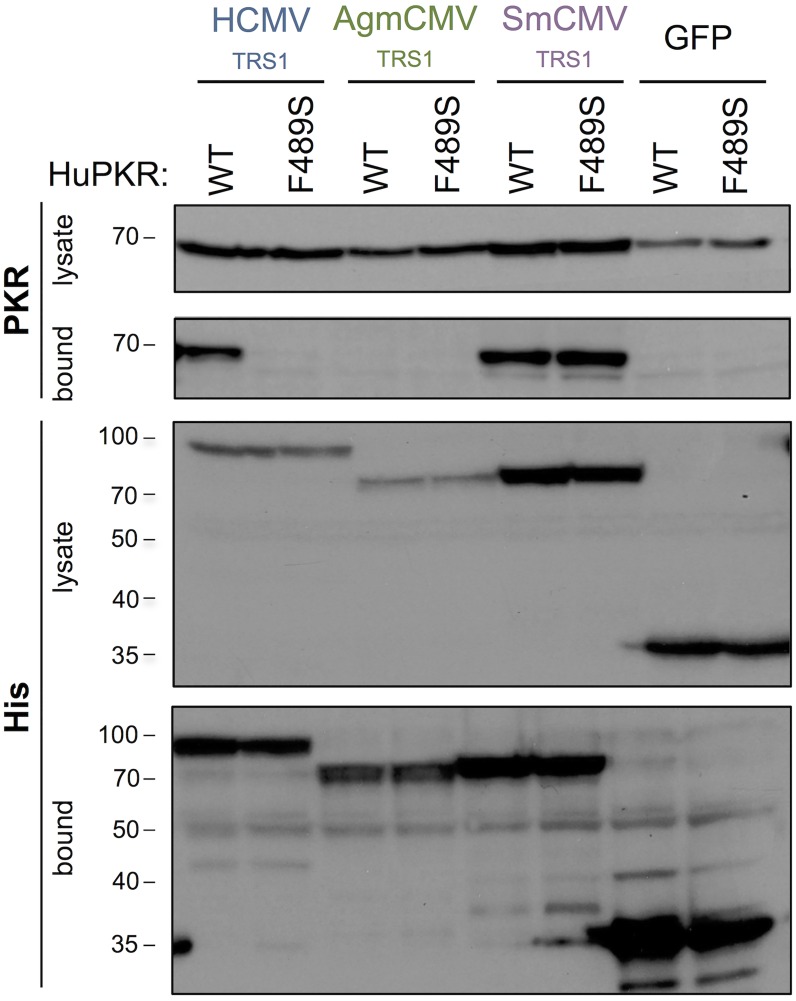
The F489S mutation eliminates HCMV_TRS1_ binding to PKR. HeLa PKR KO cells were co-transfected with WT HuPKR or HuPKR F489S and either His-tagged HCMV_TRS1_, AgmCMV_TRS1_, SmCMV_TRS1_ or EGFP. At 48h post transfection, lysates were prepared and incubated with nickel-agarose beads. Cell lysates and bound proteins were analyzed by western blotting, probing for His-tagged TRS1 proteins and for PKR. Data are representative of three independent experiments.

We next investigated how this single amino acid change made HuPKR resistant to HCMV_TRS1_. Since prior studies suggested that HCMV_TRS1_ must bind directly to PKR to effectively antagonize the PKR pathway [20–22], we tested the hypothesis that altering residue 489 of HuPKR from phenylalanine to serine interfered with HCMV_TRS1_ binding to PKR. We transfected HeLa PKR KO cells with either WT HuPKR or HuPKR F489S along with a panel of His-tagged TRS1 constructs or His-tagged GFP as a negative control. Following cell lysis, we affinity purified the His-tagged proteins along with any bound PKR. We detected TRS1 and PKR in the lysate and bound fractions using anti-His and anti-PKR antibodies, respectively (Fig 5). As expected, HCMV_TRS1_ bound to WT HuPKR; however, this interaction was severely disrupted by the F489S mutation. In contrast, SmCMV_TRS1_ bound to both WT HuPKR and the F489S mutant equally well, consistent with its ability to antagonize both forms of HuPKR (Fig 3A), while AgmCMV_TRS1_ did not bind to either PKR variant. These results indicate that position 489 is a critical residue mediating the interaction between HCMV_TRS1_ and HuPKR and that disrupting this interaction impairs HCMV_TRS1_ activity.

### Position 489 of human PKR is highly tolerant of amino acid substitutions, many of which confer resistance to antagonism by HCMV_TRS1_

**Fig 6:**
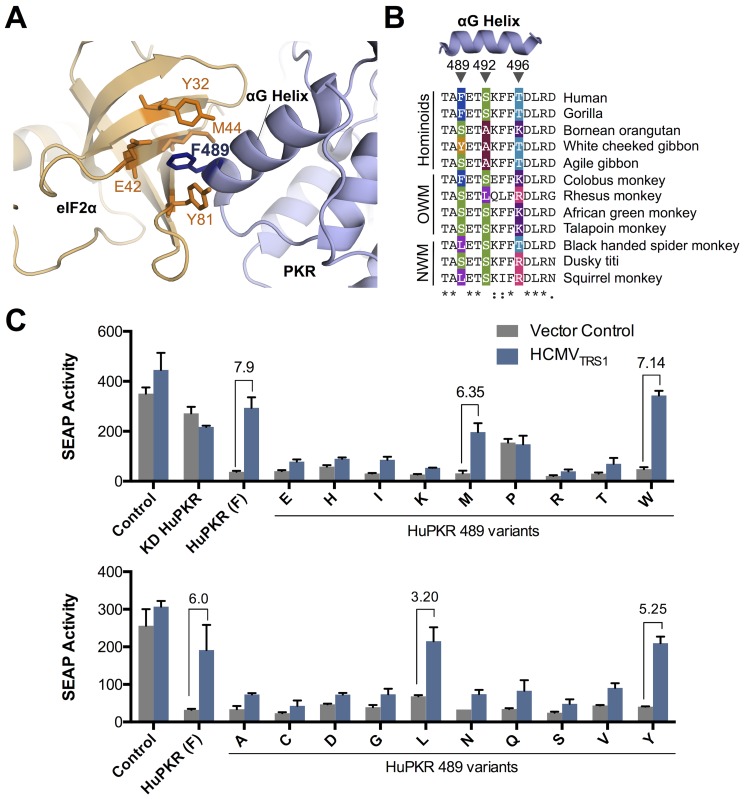
Position 489 of HuPKR is highly tolerant of amino acid substitutions. (A) Position 489 (dark blue) falls within the αG helix of PKR (light blue) and projects into a hydrophobic pocket of eIF2α (pale orange) composed of the side chains of Y32, E42, M44 and Y81 (dark orange). (B) Position 489 is highly variable in primates. The protein sequence alignment of the αG helix of PKR from representative primates is shown. Amino acids previously found to be evolving under positive selection among primates [16] are indicated with arrowheads. (C) Most mutations at position 489 retain PKR activity but only a subset are inhibited by HCMV_TRS1_. HuPKR 489 variants were evaluated as described in Fig 2B (mean ± s.d., n = 2). The fold change relative to a vector control is indicated for amino acids that are most sensitive to inhibition by HCMV_TRS1_ (greater than 3 fold increase). KD PKR is a mutant of PKR (K296R) that lacks kinase activity. Data are representative of at least two independent experiments.

Amino acid 489 falls within the αG-helix of PKR, which mediates the interaction between PKR and its downstream substrate, eIF2α [23,24]. In fact, structural data suggest that F489 directly interacts with eIF2α by projecting into a hydrophobic pocket [23] (Fig 6A). Despite this seemingly critical interaction, position 489 is rapidly evolving in primates, consistent with it being engaged in an ongoing arms race with viral antagonists [16] (Fig 6B). To determine how tolerant HuPKR function is to changes at position 489, we generated HuPKR variants by introducing all possible amino acid substitutions at position 489. These variants were then evaluated for their ability to restrict SEAP reporter gene expression, which reflects the ability of PKR to bind to and phosphorylate eIF2α and thereby inhibit translation [7]. Surprisingly, with the exception of the weak activity of proline, all of the amino acid variants maintained the ability to restrict SEAP expression to levels comparable to that of wild-type HuPKR (Fig 6C, gray bars). In contrast, a point mutant of PKR that lacks kinase activity [25] (KD HuPKR) and cannot phosphorylate eIF2α had little effect on SEAP activity. This demonstrates that, position 489 is highly tolerant of amino acid changes and suggests it does not play an essential role in the interaction between HuPKR and eIF2α. Of note, the other three cellular eIF2α kinases, which do not appear to be engaged in arms races with viral factors, have completely conserved the site corresponding to position 489 of PKR [16]. Thus, these results demonstrate the robustness of PKR’s interaction with eIF2α despite the pressure for continual innovation within the αG-helix in order to evade viral antagonists.

We next wanted to know whether HCMV_TRS1_ exhibited similar flexibility it its ability to recognize different amino acids at position 489. We found that in addition to phenylalanine, HCMV_TRS1_ could antagonize HuPKR expressing tryptophan, methionine, or tyrosine at position 489, and had moderate activity against leucine (Fig 6C). These amino acids each have hydrophobic side chains, suggesting that they may interact with a hydrophobic pocket in HCMV_TRS1_. In contrast, HCMV_TRS1_ had little activity against the other amino acid substitutions. Thus, our results clearly indicate that while HuPKR can tolerate a diverse range of amino acids at position 489 without losing its functional interaction with eIF2α, HCMV_TRS1_ is active in counteracting only a small subset of these variants. The surprising plasticity at this site may contribute to PKR’s ability to remain competitive in the arms race with rapidly evolving viral genes.

### HuPKR F489S also confers resistance to the unrelated poxvirus antagonist K3L (H47R)

**Fig 7:**
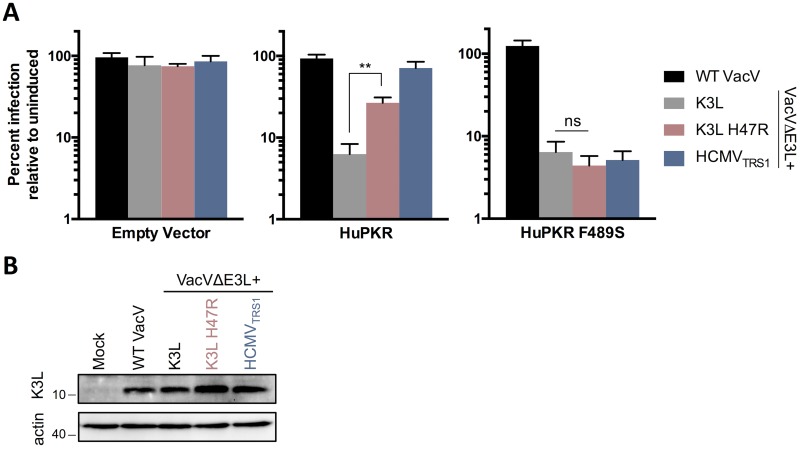
HuPKR F489S confers resistance to VacV K3L H47R. HeLa PKR KO cells inducibly expressing the indicated PKR variants were infected and evaluated as described in Fig 3A. (mean ± s.d., n = 3, ** p<0.005; ns, not significant). Data are representative of three independent experiments. (B) K3L was detected in lysates of the uninduced, infected empty vector cells from (A) by western blotting.

In addition to position 489, two other codons (492 and 496, Fig 6B) within the αG-helix are rapidly evolving among primates [16], suggesting that this helix may be a hot spot for targeting by viral antagonists. Consistent with this hypothesis, poxviruses encode a PKR pseudosubstrate that structurally mimics eIF2α and competitively inhibits eIF2α docking by binding to the αG helix. In fact, previous work on the VacV eIF2α mimic K3L identified position 492 as a major species-specific determinant of PKR activity within hominoids [16]. Another neighboring codon in human PKR, A488, has been implicated in the differential sensitivity of human vs. mouse PKR to VacV K3L [17]. The proximity of the amino acids that affect K3L and HCMV_TRS1_ activities indicate that interactions at the αG helix are essential for both antagonists. However, unlike K3L, HCMV_TRS1_ shows no obvious primary sequence homology to eIF2α, suggesting that two unrelated viruses convergently evolved to target this vulnerable region of PKR. Given the shared interaction at the αG-helix, we investigated whether altering position 489 of HuPKR would also impact the activity of K3L. WT K3L has very little activity against HuPKR, while an experimentally evolved form of K3L containing a single amino acid change (H47R) confers moderate activity against HuPKR [26,27]. Thus, we evaluated the replication profiles of viruses expressing WT K3L and K3L H47R in our HuPKR and HuPKR F489S cells. All of the viruses replicated well in the empty vector cell line and expressed similar levels of K3L (Fig 7). Similar to previous findings [26], K3L H47R rescued VACVΔE3L replication relative to WT K3L in cells expressing HuPKR (Fig 7A). Notably, the replication advantage conferred by K3L H47R was abrogated in cells expressing HuPKR F489S, demonstrating that this single amino acid change in PKR confers resistance to two unrelated viral antagonists. This result highlights the complexity of the host-virus arms race in broadly acting factors like PKR where multiple viral antagonists may target a shared interface. In these scenarios, mutations driven by one virus have the potential to alter host sensitivity or resistance to unrelated viral antagonists.

## Discussion

Our results provide evidence for a co-evolutionary history between PKR and the CMV TRS1 proteins, as reflected by specific adaptation of HCMV_TRS1_ and AgmCMV_TRS1_ to their cognate PKRs. These adaptations have led to divergence of the PKR:TRS1 interface, resulting in species-specific differences in TRS1 activity. For example, in contrast to the critical role of amino acid 489 of PKR for HCMV_TRS1_ activity, this site does not appear to mediate sensitivity to the orthologous proteins from three other primate CMVs. SmCMV_TRS1_ retains the ability to bind to and antagonize HuPKR F489S (Figs 3A and 5), and in fact it appears to be active against a broad range of primate PKRs (Fig 1A). Thus, compared to HCMV_TRS1_, SmCMV_TRS1_ may have evolved to recognize and bind to a different site that may be more conserved among primate PKRs. On the other hand, the RhCMV and AgmCMV TRS1 alleles are unable to inhibit HuPKR even when codon 489 is the variant found in their Old World monkey hosts (489S). Although we have not precisely mapped species-specific determinants in these cases, we observed that the Agm PKR kinase domain was sufficient to confer sensitivity to inhibition by AgmCMV_TRS1_ (S2A Fig). Unlike HCMV_TRS1_, which inhibits the PKR pathway prior to autophosphorylation, RhCMV_TRS1_ allows PKR autophosphorylation but blocks eIF2α phosphorylation [18]. Thus, the evolution of PKR in primates appears to have led to quite divergent adaptions in the CMV TRS1 antagonists that changed their precise binding interactions and resulted in alternative species-specific mechanisms of PKR inhibition.

Even while PKR has been evolving to evade viral antagonists, it is constrained by the need to maintain recognition of dsRNA and eIF2α. Thus, residues that are critical for these interactions are more likely to be immutable, while those that are detected by the viral antagonists are more likely to change. In fact, codons evolving under positive selection in primates were reported to be widely dispersed across the PKR coding sequences, though none were found within the dsRNA-binding domains [16]. On the other hand, at least three codons within the αG helix are highly variable among primates [16], even though the PKR kinase domain-eIF2α co-crystal shows that this region, and residue 489 in particular, makes direct contact with eIF2α [23] and thus might be expected to be particularly intolerant of substitutions. Although variation at this site in other primate PKR genes might depend on epistatic mutations elsewhere in PKR that help maintain the interaction with eIF2α, we found that HuPKR retains its inhibitory activity when any of 19 different amino acid substitutions are introduced at residue 489 in human PKR. The only exception is proline, which may not be tolerated due to its helix-breaking properties. These findings support structural data showing that the helical insert between βstrands 3 and 4 of eIF2α is relatively flexible (high B-factor) [28,29] and thus able to adapt to maintain interactions with a rapidly evolving PKR interface. In contrast, VacV K3L, the structural mimic of eIF2α, has been proposed to be more rigid and thus less tolerant of mutations in the αG helix [30]. Prior studies have identified other mutations in the αG helix that preserve (codons 490 and 499) or eliminate (codons 487 and 495) eIF2α recognition by PKR [24], but none of these are variable among primate PKR alleles. Thus, HuPKR appears to retain its eIF2α kinase function despite mutations at a subset of positions within this otherwise critical structure. This mutational tolerance at codon 489 and nearby residues may facilitate acquisition of adaptive changes in PKR during its arms race with viral antagonists that target this site.

Because PKR senses dsRNA, which accumulates during replication of diverse viruses [31] it has broad anti-viral activity and has also been the target of antagonists encoded by many different virus families [8,9]. Even within the CMV subfamily, adaptations to diverging PKR alleles during co-speciation of CMVs with their hosts have altered the PKR:TRS1 binding interfaces. In contrast to this divergent evolution of closely related PKR antagonists, here we also identify convergent evolution by the unrelated antagonists HCMV_TRS1_ and poxvirus K3L. Prior studies mapped species-specific determinants of VacV K3L sensitivity to PKR codons 488 [17] and 492 [16], both of which are within the αG helix and proximal to the site we found to be critical for sensitivity to HCMV_TRS1_. Furthermore, we demonstrate that codon 489 also impacts K3L activity, as introducing a serine at position 489 of human PKR is sufficient to confer resistance to both HCMV_TRS1_ and VacV K3L (H47R). Thus, the conflict between PKR and its viral antagonists should be viewed as a multilateral arms race, in which mutations driven by one virus can have collateral effects on other antagonists. This is true for the αG helix of PKR, which might be targeted by unrelated viral antagonists precisely because it is critical for PKR function. In response, during the evolution of host restriction factors like PKR, preservation of robustness may depend on residues, such as 489, embedded within critical functional domains that can serve as mutable decoys to enable rapid adaptation to multiple viruses without compromising the core functions of the protein.

## Materials and Methods

### Primers

All primers used to construct plasmids are listed in S1 Table.

### SEAP reporter assay and plasmids

The SEAP assay was carried out as described previously [19], except that 0.05 μg of SEAP reporter was transfected along with a given PKR and TRS1 construct at a ratio of 1:2, respectively.

The SEAP expression vector (pEQ886) has been described previously [32]. A plasmid expressing EGFP with a 6x-His tag (pEQ1100) was used as a the control and vector control in SEAP assays and has been described previously [33]. HCMV_TRS1_ was expressed from pEQ1180 (formerly 981) [34]. AgmCMV_TRS1_ was PCR amplified from AgmCMV DNA (ATTC VR-706) using primers 931 and 932 and TOPO cloned into pcDNA3.1V5-6xHis (ThermoFischer Scientific) to yield pEQ1377. RhCMV_TRS1_ was digested from pEQ1215 [18] with HindIII and NotI and ligated into pEQ1180 that had been cut with the same enzymes to yield pEQ1261. Because we were unsuccessful in PCR amplifying SmCMV_TRS1_ from viral DNA, possibly due to its very high GC content, we synthesized a mammalian codon-optimized form of SmCMV_TRS1_ flanked by the MCS from pcDNA3.1v5-His to facilitate later cloning (GenScript Inc.; GenBank accession number KX518569). This synthesized construct was inserted into pUC57 using EcoRI and HindIII to produce pEQ1494. SmCMV_TRS1_ was removed from pEQ1494 using Asp718 and NotI and ligated into pEQ1377 cut with the same enzymes, resulting in pEQ1495, which was used in SEAP experiments.

A knockdown resistant form of HuPKR (SR#329) generously provided by Stefan Rothenburg (Kansas State University) [17] was used for HuPKR expression in Figs 2B and 6C. To generate a construct expressing active HuPKR with a 6xHis tag, pSB819+HuPKR [16] was digested with EcoRI to yield a PKR fragment that was then ligated into the same sites in pEQ1198 [18], resulting in pEQ1356. However, PKR expression from pEQ1356 was insufficient to repress SEAP expression in the reporter assay, so HuPKR with a 6xHis tag was then PCR amplified from pEQ1356 using primers 2058 and 2059, and cloned into SR#329 cut with KpnI and HindIII using Gibson assembly, resulting in pEQ1563. Similarly, AgmPKR was isolated from pSB819+AgmPKR, which has been described previously [16], and introduced into a pcDNA3.1v5-His backbone that also contained a biotinylation signal between XhoI and XbaI, resulting in pEQ1357. AgmPKR was PCR amplified from pEQ1357 using primers 2058 and 2059 and introduced into SR#329 that had been digested with KpnI and HindIII using Gibson assembly (New England Biolabs) to produce pEQ1564. pEQ1563 and pEQ1564 were used in S2A Fig.

To generate chimeras between HuPKR and AgmPKR, the N-terminal half of HuPKR or AgmPKR was amplified from pEQ1356 and pEQ1357, respectively, using primers 2033 and 2036, while the C-terminal half of each PKR was amplified using primers 2034 and 2035. The N-terminal and C-terminal fragments were then cloned into pEQ1357 digested with BamHI and EcoRV using Gibson assembly to yield a Hu-Agm chimera (pEQ1555) and an Agm-Hu chimera (pEQ1556) in the pcDNA3.1v5-His backbone. The two his-tagged chimeras were then PCR-amplified from these constructs using primers 2058 and 2059 and inserted into SR#329 digested with KpnI and HindIII using Gibson assembly, resulting in pEQ1571 (Hu-Agm PKR-6xHis) and pEQ1572 (Agm-Hu PKR-6xHis), which were used in S2A Fig. Constructs without tags were generated as follows: HuPKR and AgmPKR were PCR amplified from pEQ1356 and pEQ1357 using primers 2102 and 2104 or 2102 and 2103, respectively. The PCR products were then introduced into SR#329 cut with KpnI and HindIII by Gibson assembly to yield pEQ1602 (HuPKR) and pEQ1598 (AgmPKR), which were used in Fig 2C and S2B Fig.

To generate the Hu-AgmD chimera, the N-terminal portion of HuPKR was PCR amplified from pEQ1356 using primers 2102 and 2085, while the AgmD fragment was amplified from pEQ1357 using primers 2084 and 2103. These fragments were cloned into SR#329 using Gibson assembly, resulting in pEQ1600. The Hu-AgmD1, D2 and D3 chimeras were first constructed with 6xHis tags by PCR amplifying the various regions of HuPKR and AgmPKR as follows. Hu-AgmD1: pEQ1356 was PCR-amplified using primer pairs 2058, 2085 and 2098, 2059; pEQ1357 was PCR amplified using primers 2084, 2099. Hu-AgmD2: pEQ1356 was PCR-amplified using primer pairs 2058, 2099 and 2100, 2059; pEQ1357 was PCR amplified using primers 2098, 2101. Hu-AgmD3: pEQ1356 was PCR amplified using primers 2058, 2101; pEQ1357 was PCR amplified using primers 2100, 2059. The fragments were then cloned into SR#329 using Gibson assembly, resulting in pEQ1595 (Hu-AgmD1-His), pEQ1596 (Hu-AgmD2-His), and pEQ1597 (Hu-AgmD3-His). To remove the 6xHis tags, the chimeras were PCR-amplified using the following primers: 2102, 2104 (pEQ1595 and pEQ1596) or 2102, 2103 (pEQ1597). The PCR products were cloned into SR#329 to generate pEQ1603 (Hu-AgmD1), pEQ1604 (Hu-AgmD2) and pEQ1599 (Hu-AgmD3), which were used in S2B Fig.

Point mutations in HuPKR were constructed by using complementary forward and reverse primers harboring the desired mutation paired with primers at the beginning or end of PKR, as shown in S2 Table. pEQ1356 was used as a template for PCR amplification and the resulting N and C terminal fragments containing the desired mutations were cloned into SR#329 cut with KpnI and HindIII of using Gibson assembly.

### VacV recombinant viruses and infections

WT VacV (VC2+lacZ), VacVΔE3L, VacVΔE3L+HCMV_TRS1_ (VVeq1148), and VacVΔE3L+RhCMV_TRS1_ (VVeq1233) have been described previously [18]. To generate VacVΔE3L+AgmCMV_TRS1_, AgmCMV_TRS1_ was first removed from pEQ1377 (see SEAP plasmids, above) using Asp718 and NotI and ligated into pEQ1233 [18], that had been digested with the same enzymes, resulting in pEQ1453. pEQ1453 was then used to introduce AgmCMV_TRS1_ into the thymidine kinase locus in VacVΔE3L through homologous recombination to generate VacVΔE3L+AgmCMV_TRS1_ (VVeq1453). Similarly, SmCMV_TRS1_ was cut from pEQ1495 (see SEAP plasmids) with Asp718 and NotI and inserted into the same sites of pEQ1453 to produce pEQ1497, which was recombined into VacVΔE3L to produce VacVΔE3L+SmCMV_TRS1_ (VVeq1497). Recombinant VacVs were propagated and titered in BHK cells.

In all viral replication experiments, cells were infected at an MOI of 0.1 for 1 hour, after which the medium was replaced. Viral replication was evaluated at 48 hpi by measuring β-Galactosidase (β-Gal) activity via a fluorometric substrate cleavage assay, as described previously [33], or through plaque assays in HeLa PKR KO cells (S1B Fig). With the stable cell lines expressing PKR in an inducible manner (Figs 3 and 7), cells were treated with medium containing 1 μg/ml of doxycycline 24 hours prior to infection, infected in the absence of doxycycline, and refed with medium containing 1ug/ml of doxycycline after the hour-long infection period.

To evaluate the status of PKR phosphorylation during infection, cells were treated with medium containing 1 μg/ml of doxycycline for 24 hours and then infected at an MOI of 3 for 1 hour, after which the cells were re-fed with medium containing 1 μg/ml of doxycycline. Cell lysates were harvested in 2% SDS at 6 hpi.

### Cell Lines

HeLa, BSC40, and HeLa PKR KO cell lines were maintained at 37°C with 5% CO_2_ in Dulbecco’s modified Eagle’s medium (DMEM) supplemented with 10% NuSerum (BD Biosciences). HeLa cells were provided by Bertram Jacobs (Arizona State University) and BSC40 cells were provided by Stanley Riddell (Fred Hutchinson Cancer Research Center, Seattle, WA).

HeLa PKR KO cell lines were generated by transfecting HeLa cells with plasmid vectors that express Cas9 (HCas9, a gift from George Church, Addgene #41815 [35], two guide RNAs (pEQ 1451 and pEQ1452) that target genomic sequences upstream (5’-TCTCTTCCATTGTAGGATA-3’) and downstream (5’-CTTTTCTTCCACACAGTCA-3’) of PKR, and a homologous recombination vector containing mCherry and puromycin (pEQ1489). After puromycin selection, single-cell clones were evaluated by sequencing and immunoblotting to identify clean knockouts; Clone #6 was used in these studies.

To generate stable cell lines expressing different PKR constructs in the HeLa PKR KO cell line, we first cloned each PKR into the pSLIK-Hygro lentiviral vector (a gift from Iain Fraser, Addgene plasmid # 25737 [36]). HuPKR was PCR amplified from pEQ1356 using primers 2105 and 2106 and inserted into pEN_TmiRc3 Entry Vector (a gift from Iain Fraser, Addgene plasmid # 25748 [36]) at sites SpeI and XbaI using Gibson assembly to produce pEQ1606. HuPKR was then moved from pEQ1606 into the pSLIK-Hygro destination vector using Gateway Cloning (Thermo Fisher Scientific), resulting in pEQ1607. To move AgmPKR into this vector, AgmPKR was PCR amplified from pEQ1357 using primers 2175 and 2176, and inserted into pEQ1607 that had been digested with BstEII using Gibson assembly, yielding pEQ1641. Similarly, HuPKR F489S was PCR-amplified from pEQ1624 using primers 2173 and 2174 and introduced into pEQ1607 that had been digested with BstEII using Gibson assembly, resulting in pEQ1642. HeLa PKR KO cells were transduced with lentiviral vectors encoding pEQ1607, pEQ1641, pEQ1642, and pSLIK-Hygro (empty vector). After hygromycin selection, single cell clones were evaluated for their ability to restrict VacVΔE3L and for PKR expression levels, with the following clones used in experiments: HeLa PKR KO+1607#9, 1641#3 and 1642#2.

### Immunoblot Assays

Cell lysates were harvested in 2% sodium dodecyl sulfate (SDS) and equal amounts of lysates were separated on a 10% SDS-polyacrylamide gel (except for Fig 7, where a 15% SDS-polyacrylamide gel was used) and transferred to polyvinylide difluoride (PVDF) membranes. Proteins were detected using the Western-Star chemiluminescent detection system (Applied Biosystems) with the following primary antibodies: PKR D7F7 (#12297, Cell Signaling Technology), P-PKR E120 (ab32036, abcam), Penta-His (34660, Qiagen), K3L (a gift from J. Tartaglia [37]), P-eIF2α (#3597, Cell Signaling), total eIF2α (#2103, Cell Signaling), and Actin (A2066, Sigma).

### Pulldown assays

HeLa PKR KO cells were transfected using Lipofectamine 2000 (Invitrogen) with 0.15 μg of HuPKR (pEQ1602) or HuPKR F489S (pEQ1624) and 2.35μg of HCMV_TRS1_ (pEQ1180), AgmCMV_TRS1_ (pEQ1377), SmCMV_TRS1_ (pEQ1495) or EGFP (pEQ1100). At 48 hours post-transfection, cells were washed with PBS and harvested in 250μl of cold NiNTA Lysis Buffer (50mM NaH2PO4, 300mM NaCl, 10mM imidazole, 0.75% Tween 20, 1μM benzamidine and 100 μM PMSF). Lysates were incubated on ice for 20 minutes with occasional vortexing and centrifuged at 16,000xg for 10 min at 4°C to remove cell debris. 20 μl of each lysate was reserved, while 200 μl of the remaining lysate was added to 30 μl of PerfectPro NiNTA Superflow agarose (5prime) and incubated at 4°C on a rotating mixer for 2 hours. After binding, the beads were pelleted and washed 3x with 500μl of NiNTA Wash buffer (50mM NaH2PO4, 300mM NaCl, 20mM imidazole, 0.75% Tween 20). The lysate and bound samples were denatured in SDS-PAGE sample buffer at 95°C (5 minutes) and separated on a 10% SDS-polyacrylamide gel, transferred to a PVDF membrane and probed with PKR D7F7 (Cell Signaling Technology) and Penta-His (Qiagen) antibodies.

### Structure observations and protein alignments

The structure of PKR in complex with eIF2α was visualized using data from the protein databank (http://www.pdb.org; ID 2A1A) and MacPyMol.

For sequence comparisons of the αG helix among primate PKRs, the following sequences were obtained from NCBI and aligned using Clustal Omega: *Homo sapiens* (human; BC093676), *Gorilla gorilla* (Gorilla; EU733258), *Pongo pygmaeus pygmaeus* (Bornean orangutan; EU733259), *Nomascus leucogenys* (northern white-cheeked gibbon; EU733257), *Hylobates agilis albibarbis* (Agile gibbon; EU733270), *Colobus guereza* (Colobus monkey; EU733267), *Macaca mulatta* (Rhesus monkey; EU733261), *Cercopithecus aethiops* (African green monkey; EU733254), *Miopithecus talapoin talapoin* (Talapoin monkey; EU733269), *Ateles geoffroyi* (Black-handed spider monkey; EU733263), *Callicebus moloch* (Dusky titi; EU733265), *Saimiri boliviensis boliviensis* (Squirrel monkey, XM_003926814).

### Nucleotide sequence accession numbers

The sequence of the mammalian codon-optimized SmCMV_TRS1_ has been deposited in GenBank under the following accession number: KX518569.

## Supporting Information

S1 Fig VacV replication strongly correlates with β-gal activity. (A) HeLa PKR KO, HeLa (human), or BSC40 (Agm) cells were infected (MOI 0.1) with WT VacV, VacVΔE3L, or VacVΔE3L recombinants containing HCMV_TRS1_, AgmCMV_TRS1_, RhCMV_TRS1_, or SmCMV_TRS1_. At 48 hpi, β-gal activity was measured (mean ± s.d.). (B) VacV titers of freeze-thaw lysates from (A) were determined by plaque assays in HeLa PKR KO cells (mean ± s.d.).
(TIFF)

S2 Fig Mapping of the molecular determinant of sensitivity of HuPKR to HCMV_TRS1_. (A) PKR resistance maps to the kinase domain. Chimeras generated between HuPKR and AgmPKR are shown. The chimeras were evaluated as described in Fig 2B (mean ± s.d.). In this experiment, all PKR constructs contained C-terminal epitope tags, which were not used in other experiments as we discovered that the tags attenuated the inhibitory effect of PKR. (B) Resistance to HCMV_TRS1_ maps to the D2 region, codons 475–520, of Agm PKR. The kinase domain was subdivided to create additional chimeras, which were evaluated as described in Fig 2B (mean ± s.d.).
(TIF)

S1 Table List of primers used in materials and methods. (DOCX)

S2 Table Sets of primers used to construct point mutants. (DOCX)
