# Supplementary figures and images for: A Single Amino Acid Dictates Protein Kinase R Susceptibility to Unrelated Viral Antagonists
Source: PLoS Pathog. 2016 Oct 25;12(10):e1005966. doi: 10.1371/journal.ppat.1005966 (PMC5079575; doi:10.1371/journal.ppat.1005966)

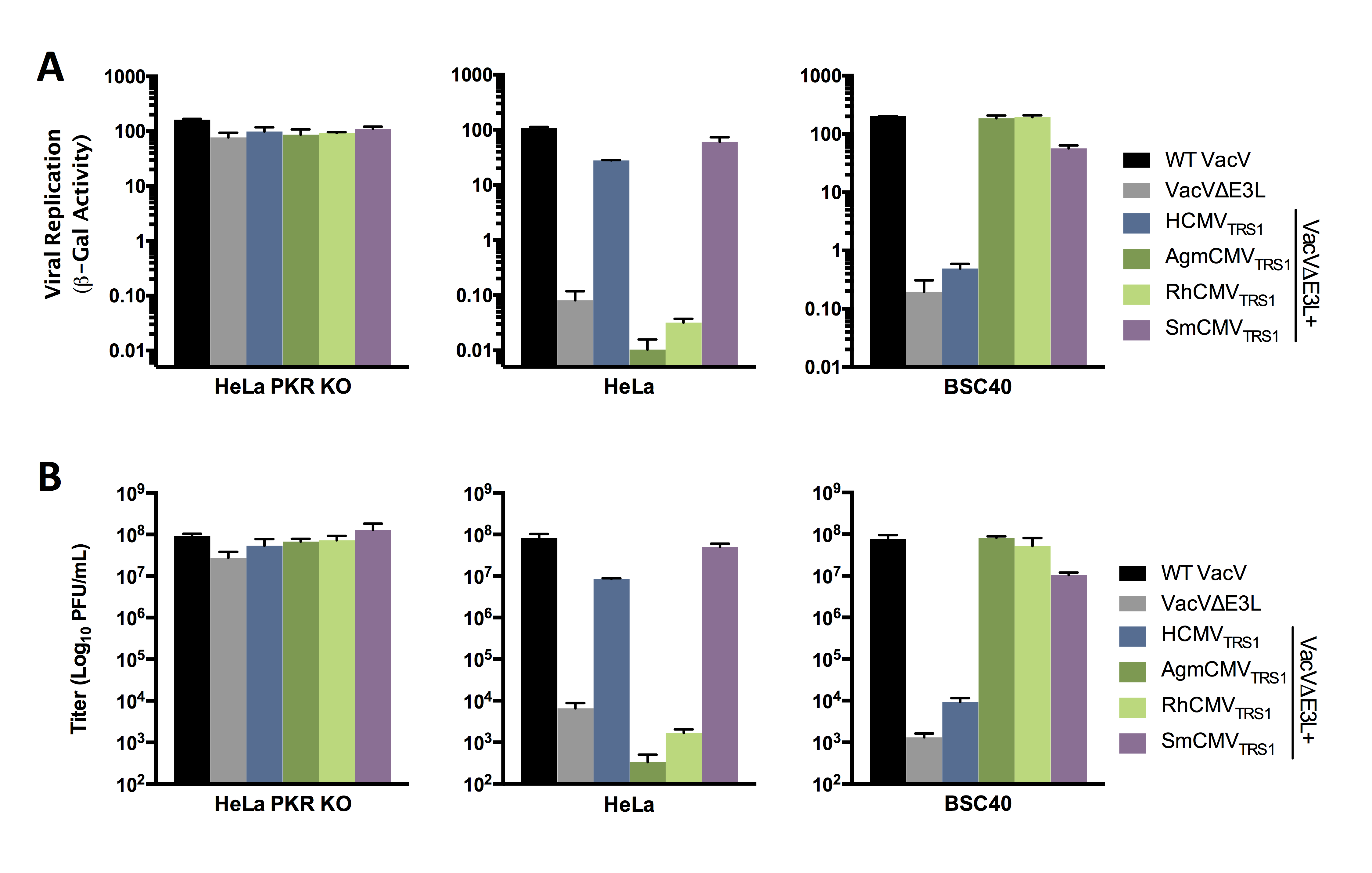

Supplement: S1 Fig — (A) HeLa PKR KO, HeLa (human), or BSC40 (Agm) cells were infected (MOI 0.1) with WT VacV, VacVΔE3L, or VacVΔE3L recombinants containing HCMVTRS1, AgmCMVTRS1, RhCMVTRS1, or SmCMVTRS1. At 48 hpi, β-gal activity was measured (mean ± s.d.). (B) VacV titers of freeze-thaw lysates from (A) were determined by plaque assays in HeLa PKR KO cells (mean ± s.d.). (TIFF) [file ppat.1005966.s001.tiff]

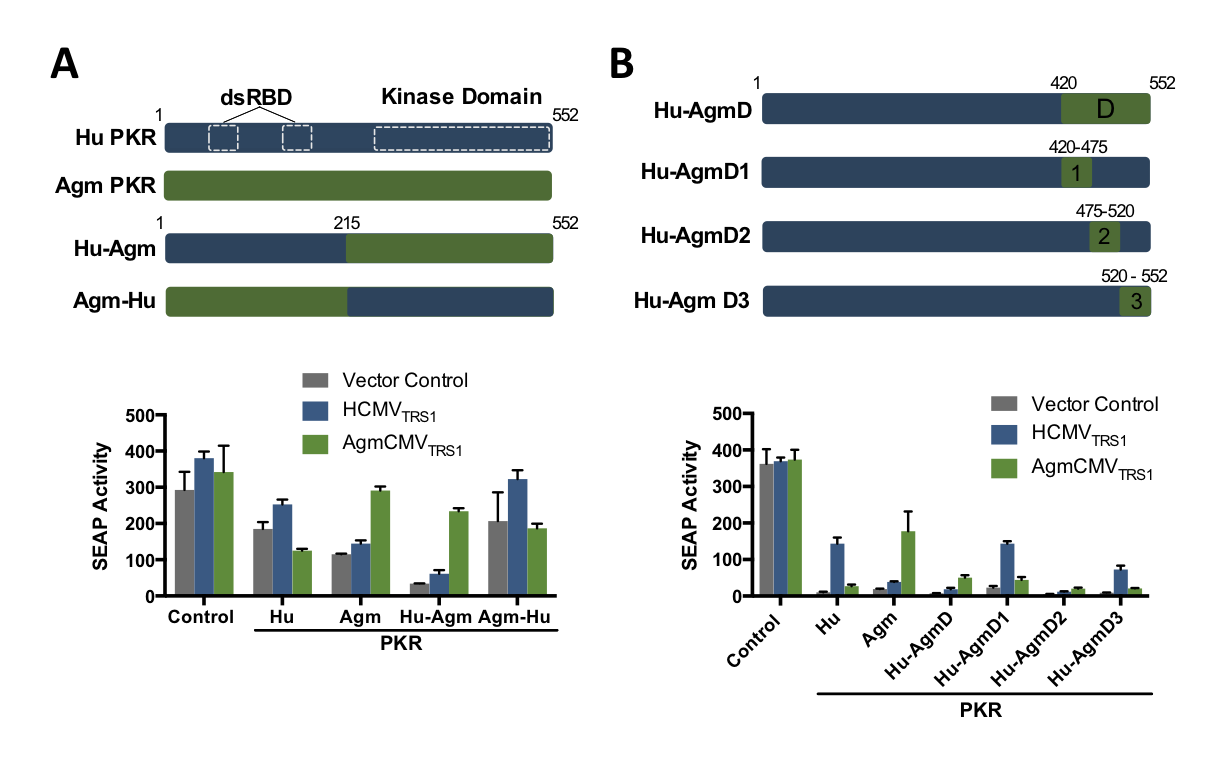

Supplement: S2 Fig — (A) PKR resistance maps to the kinase domain. Chimeras generated between HuPKR and AgmPKR are shown. The chimeras were evaluated as described in Fig 2B (mean ± s.d.). In this experiment, all PKR constructs contained C-terminal epitope tags, which were not used in other experiments as we discovered that the tags attenuated the inhibitory effect of PKR. (B) Resistance to HCMVTRS1 maps to the D2 region, codons 475–520, of Agm PKR. The kinase domain was subdivided to create additional chimeras, which were evaluated as described in Fig 2B (mean ± s.d.). (TIF) [file ppat.1005966.s002.tif]
