# Supplementary material for: A Single Amino Acid Dictates Protein Kinase R Susceptibility to Unrelated Viral Antagonists
Source: PLoS Pathog. 2016 Oct 25;12(10):e1005966. doi: 10.1371/journal.ppat.1005966 (PMC5079575; doi:10.1371/journal.ppat.1005966)
Supplement: S1 Table — (DOCX) [file ppat.1005966.s003.docx]

**Table S1. List of primers used in materials and methods.**

| **Primer number** | **Sequence (5' to 3')** |
| --- | --- |
| 931 | ACCACCATGACCGGCCGCAGACCTCCGCGC |
| 932 | ACAGGGAATGACGAGCACCCCGTCGCTGCT |
| 2033 | GTT AAG CTT GGT ACC GAG CTC G |
| 2034 | GGC CGC CAC TGT GCT GGA T |
| 2035 | GCA GAT ACA TCA GAG ATA AAT TCT AAC AG |
| 2036 | CTG TTA GAA TTT ATC TCT GAT GTA TCT GC |
| 2058 | AAT TCG AGC TCG GTA CGC TAG TTA AGC TTG GTA CC |
| 2059 | TAA TAA GAT CTC TCG AGA GGG TTT AAA CTC AAT GGT G |
| 2084 | CAT AGA GAT CTT AAG CCA AGT AAT ATA TTC |
| 2085 | GAA TAT ATT ACT TGG CTT AAG ATC TCT ATG |
| 2098 | GAA GTG GAC CTC TAC GCT TTG G |
| 2099 | CCA AAG CGT AGA GGT CCA CTT C |
| 2100 | AGA AAT TAC TCT CAA AGA AAC CTG AGG |
| 2101 | CCT CAG GTT TCT TTG AGA GTA ATT TCT |
| 2102 | AAT TCG AGC TCG GTA CCA TGG CTG GTG ATC TT |
| 2103 | TAA TAA GAT CTC TCG AGA AGC TTC TAA CAT GTA TGT CGT TCC TTT TT |
| 2104 | TAA TAA GAT CTC TCG AGA AGC TTC TAA CAT GTG TGT CGT TCA TT |
| 2105 | CCT AGG CGT CTG ATC ACT AGT GGT AAC CAT GGC TGG TGA TCT TTC AG |
| 2106 | GTA CAA GAA AGC TGG GTC TAG AGG TGA CCC TAA CAT GTG TGT CGT TCA TT |
| 2128 | ATG TGA CAC TGC TTC CGA AAC ATC AAA GTT |
| 2129 | AAC TTT GAT GTT TCG GAA GCA GTG TCA CAT |
| 2130 | ATC AAA GTT TTT CAA AGA CCT ACG GGA TG |
| 2131 | CAT CCC GTA GGT CTT TGA AAA ACT TTG AT |
| 2132 | TAC GGG ATG GCA CCA TCT CAG ATA TAT |
| 2133 | ATA TAT CTG AGA TGG TGC CAT CCC GTA |
| 2134 | GCA TCA TCT CAG ATG TGT TTG ATA AAA AAG A |
| 2135 | TCT TTT TTA TCA AAC ACA TCT GAG ATG ATG C |
| 2136 | TAT ATT TGA TAA AAG AGA AAA AAC TCT TC |
| 2137 | GAA GAG TTT TTT CTC TTT TAT CAA ATA TA |
| 2138 | GAA AAA ACT CTT CTA GAG AAA TTA CTC TCA |
| 2139 | TGA GAG TAA TTT CTC TAG AAG AGT TTT TTC |
| 2167 | ATG TGA CAC TGC TTA TGA AAC ATC AAA GTT |
| 2168 | AAC TTT GAT GTT TCA TAA GCA GTG TCA CAT |
| 2169 | ATG TGA CAC TGC TTT GGA AAC ATC AAA GTT |
| 2170 | AAC TTT GAT GTT TCC AAA GCA GTG TCA CAT |
| 2173 | CGT CTG ATC ACT AGT GGT AAC CAT GGC TGG TGA TCT TTC AG |
| 2174 | AAA GCT GGG TCT AGA GGT CAC CCT AAC ATG TGT GTC GTT C |
| 2175 | CGT CTG ATC ACT AGT GGT AAC CAT GGC TGG TGA TCT TGC AC |
| 2176 | AAA GCT GGG TCT AGA GGT CAC CCT AAC ATG TAT GTC GTT C |
| 2198 | ATG TGA CAC TGC TAT CGA AAC ATC AAA GTT |
| 2199 | AAC TTT GAT GTT TCG ATA GCA GTG TCA CAT |
| 2200 | AGA CTT ACG TTA TTA GAC GTG TTA AAT ATA A |
| 2201 | TTA TAT TTA ACA CGT CTA ATA ACG TAA GTC T |
| 2206 | GTA TGT GAC ACT GCT TGG GAA ACA TCA AAG TTT |
| 2207 | AAA CTT TGA TGT TTC CCA AGC AGT GTC ACA TAC |
| 2208 | GTA TGT GAC ACT GCT GAG GAA ACA TCA AAG TTT |
| 2209 | AAA CTT TGA TGT TTC CTC AGC AGT GTC ACA TAC |
| 2210 | GTA TGT GAC ACT GCT CAC GAA ACA TCA AAG TTT |
| 2211 | AAA CTT TGA TGT TTC GTG AGC AGT GTC ACA TAC |
| 2212 | GTA TGT GAC ACT GCT ATG GAA ACA TCA AAG TTT |
| 2213 | AAA CTT TGA TGT TTC CAT AGC AGT GTC ACA TAC |
| 2214 | GTA TGT GAC ACT GCT CCC GAA ACA TCA AAG TTT |
| 2215 | AAA CTT TGA TGT TTC GGG AGC AGT GTC ACA TAC |
| 2216 | GTA TGT GAC ACT GCT CGG GAA ACA TCA AAG TTT |
| 2217 | AAA CTT TGA TGT TTC CCG AGC AGT GTC ACA TAC |
| 2218 | GTA TGT GAC ACT GCT AAG GAA ACA TCA AAG TTT |
| 2219 | AAA CTT TGA TGT TTC CTT AGC AGT GTC ACA TAC |
| 2220 | GTA TGT GAC ACT GCT GAC GAA ACA TCA AAG TTT |
| 2221 | AAA CTT TGA TGT TTC GTC AGC AGT GTC ACA TAC |
| 2222 | GTA TGT GAC ACT GCT AAC GAA ACA TCA AAG TTT |
| 2223 | AAA CTT TGA TGT TTC GTT AGC AGT GTC ACA TAC |
| 2224 | GTA TGT GAC ACT GCT CAG GAA ACA TCA AAG TTT |
| 2225 | AAA CTT TGA TGT TTC CTG AGC AGT GTC ACA TAC |
| 2226 | GTA TGT GAC ACT GCT TGC GAA ACA TCA AAG TTT |
| 2227 | AAA CTT TGA TGT TTC GCA AGC AGT GTC ACA TAC |
| 2228 | GTA TGT GAC ACT GCT GGC GAA ACA TCA AAG TTT |
| 2229 | AAA CTT TGA TGT TTC GCC AGC AGT GTC ACA TAC |
| 2230 | GTA TGT GAC ACT GCT GCC GAA ACA TCA AAG TTT |
| 2231 | AAA CTT TGA TGT TTC GGC AGC AGT GTC ACA TAC |
| 2232 | GTA TGT GAC ACT GCT GTG GAA ACA TCA AAG TTT |
| 2233 | AAA CTT TGA TGT TTC CAC AGC AGT GTC ACA TAC |
| 2234 | GTA TGT GAC ACT GCT ACC GAA ACA TCA AAG TTT |
| 2235 | AAA CTT TGA TGT TTC GGT AGC AGT GTC ACA TAC |
|  |  |
