# Supplementary material for: A Single Amino Acid Dictates Protein Kinase R Susceptibility to Unrelated Viral Antagonists
Source: PLoS Pathog. 2016 Oct 25;12(10):e1005966. doi: 10.1371/journal.ppat.1005966 (PMC5079575; doi:10.1371/journal.ppat.1005966)
Supplement: S2 Table — (DOCX) [file ppat.1005966.s004.docx]

| **S2 Table. Sets of primers used to construct point mutants.** | | | |
| --- | --- | --- | --- |
|  |  |  |  |
| HuPKR Mutation | pEQ number | N-terminal  fragment primers | C-terminal  fragment primers |
| K296R | 1645 | 2102, 2201 | 2104, 2200 |
| F489S | 1624 | 2102, 2129 | 2104, 2128 |
| T496K | 1625 | 2102, 2131 | 2104, 2130 |
| I502T | 1626 | 2102, 2133 | 2104, 2132 |
| I506V | 1627 | 2102, 2135 | 2104, 2134 |
| K510R | 1628 | 2102, 2137 | 2104, 2136 |
| Q516E | 1629 | 2102, 2139 | 2104, 2138 |
| F489Y | 1633 | 2102, 2168 | 2104, 2167 |
| F489L | 1631 | 2102, 2170 | 2104, 2169 |
| F489I | 1646 | 2102, 2199 | 2104, 2198 |
| F489E | 1647 | 2102, 2209 | 2104, 2208 |
| F489W | 1648 | 2102, 2207 | 2104, 2206 |
| F489H | 1649 | 2102, 2211 | 2104, 2210 |
| F489M | 1650 | 2102, 2213 | 2104, 2212 |
| F489P | 1651 | 2102, 2215 | 2104, 2214 |
| F489T | 1652 | 2102, 2235 | 2104, 2234 |
| F489R | 1655 | 2102, 2217 | 2104, 2216 |
| F489K | 1656 | 2102, 2219 | 2104, 2218 |
| F489D | 1657 | 2102, 2221 | 2104, 2220 |
| F489Q | 1658 | 2102, 2225 | 2104, 2224 |
| F489A | 1659 | 2102, 2231 | 2104, 2230 |
| F489G | 1662 | 2102, 2229 | 2104, 2228 |
| F489V | 1663 | 2102, 2233 | 2104, 2232 |
| F489N | 1666 | 2102, 2223 | 2104, 2222 |
|  |  |  |  |
